# The role of neural connexins in HeLa cell mobility and intercellular communication through tunneling tubes

**DOI:** 10.1186/s12860-016-0080-1

**Published:** 2016-01-13

**Authors:** Lina Rimkutė, Vaidas Jotautis, Alina Marandykina, Renata Sveikatienė, Ieva Antanavičiūtė, Vytenis Arvydas Skeberdis

**Affiliations:** Institute of Cardiology, Lithuanian University of Health Sciences, 17 Sukilėlių Ave., 50009 Kaunas, Lithuania

**Keywords:** Tunneling tubes, Connexins, Gap junction channels, Cell mobility, siRNA transport

## Abstract

**Background:**

Membranous tunneling tubes (TTs) are a recently discovered new form of communication between remote cells allowing their electrical synchronization, migration, and transfer of cellular materials. TTs have been identified in the brain and share similarities with neuronal processes. TTs can be open-ended, close-ended or contain functional gap junctions at the membrane interface. Gap junctions are formed of two unapposed hemichannels composed of six connexin (Cx) subunits. There are evidences that Cxs also play channel-independent role in cell adhesion, migration, division, differentiation, formation of neuronal networks and tumorigenicity. These properties of Cxs and TTs may synergetically determine the cellular and intercellular processes. Therefore, we examined the impact of Cxs expressed in the nervous system (Cx36, Cx40, Cx43, Cx45, and Cx47) on: 1) cell mobility; 2) formation and properties of TTs; and 3) transfer of siRNA between remote cells through TTs.

**Results:**

We have identified two types of TTs between HeLa cells: F-actin rich only and containing F-actin and α-tubulin. The morphology of TTs was not influenced by expression of examined connexins; however, Cx36-EGFP-expressing cells formed more TTs while cells expressing Cx43-EGFP, Cx45, and Cx47 formed fewer TTs between each other compared with *wt* and Cx40-CFP-expressing cells. Also, Cx36-EGFP and Cx40-CFP-expressing HeLa cells were more mobile compared with *wt* and other Cxs-expressing cells. TTs containing Cx40-CFP, Cx43-EGFP, or Cx47 gap junctions were capable of transmitting double-stranded small interfering RNA; however, Cx36-EGFP and Cx45 were not permeable to it. In addition, we show that Cx43-EGFP-expressing HeLa cells and laryngeal squamous cell carcinoma cells can couple to the mesenchymal stem cells through TTs.

**Conclusions:**

Different Cxs may modulate the mobility of cells and formation of TTs in an opposite manner; siRNA transfer through the GJ-containing TTs is Cx isoform-dependent.

**Electronic supplementary material:**

The online version of this article (doi:10.1186/s12860-016-0080-1) contains supplementary material, which is available to authorized users.

## Background

Directed cell migration is a pivotal process for normal development and morphogenesis of most animals, wound healing, tissue renewal, immune responses, angiogenesis, and tumor metastasis [1, 2]. During these processes, cells are subjected to stress and increased energy demands. A growing body of evidence suggests that a newly discovered form of intercellular communication referred to as “intercellular bridges” or “tunneling nanotubes” and “tunneling tubes” (TTs) contributes to cell movement [3–5] and provides means for energy supply to the remote cells by transporting ATP and even mitochondria [5–7]. Basically, TTs form when filopodial or lamellipodial protrusions from one cell attach to the target cell or during dislodgement of abutted cells [8]. In these ways, remote cells can establish open-ended, close-ended, or gap junction (GJ)-based communication. TTs have been shown to be implicated in the intercellular electrical coupling and Ca^2+^ flux; transfer of organelles or proteins; virus, pathogenic prion, and protein transmission; cell migration; and bacteria capture (reviewed in refs. [8–11]).

Recently, it has been proposed that connexins (Cxs), in addition to their canonical function of composing GJs, play a channel-independent role in cell adhesion, migration, division, differentiation, and tumorigenicity (reviewed in refs. [12–14]). Among 21 isoforms of Cxs found in the human genome, the role of only Cx26, Cx31.1, Cx32, and Cx43 in these processes has been described in the scientific literature so far [14]. It has been shown that Cx26 inhibits cell migration by altering the distribution of actin filaments; Cx31.1 decreases cell proliferation, delays the cell cycle at the G1 phase, and decreases migration and invasion of lung cancer cells; Cx32 increases cell proliferation, migration, and invasion; Cx43 increases cell migration, induces actin cytoskeleton reorganization, and reduces cell proliferation. Cxs interacting with cytoskeletal and tight junction proteins [12, 15] contribute to the regulation of cell migration, directed outgrowing of filopodial and lamellipodial protrusions [12, 16–18], and intercellular communication through TTs [5].

Eleven isoforms of Cxs have been identified in the nervous system where they can play an important role in the directed migration of cells, formation of neural processes, and progression of brain tumors [19]. Astrocytes express high levels of Cxs and can couple to neurons and oligodendrocytes. Astrocyte dysfunction may cause neuroautoimmune diseases, neoplasms, and epilepsy [20]. Neuronal processes share structural and functional similarities with TTs, and the directed formation of TTs between developing neurons and astrocytes has been demonstrated [21]. Cx43 accumulation at the tips of filopodium-like structures of astrocytes [22] may cause more frequent filopodium formation [23], stabilization of the leading edge protrusions in neuronal cells [24], and biological molecule transmission via TT-like structures [25]. Thus, TTs and GJs in the brain are likely to facilitate the intercellular exchange of materials and possibly genetic information. The last may be of particular importance in determining the stem cell differentiation, cancer invasion, and metastasis. The role of Cxs in cancer is controversial as well as tissue- and cancer stage-specific. Reduced Cx expression, or redistribution from the membrane to the cytoplasm, has been documented in a variety of cancers, including colon, lung, ovarian, breast, endometrial, and renal cell carcinomas and sarcomas, gliomas as well as in pre-cancerous tissues such as that of cervix. Up-regulated Cx expression has also been frequently described, and examples include breast cancer, skin cancers and various squamous cell carcinomas, colon cancer, and pancreatic cancer. Even within the same tumor type, both increased and decreased Cx expression can be found (reviewed in ref. [26]). In gliomas, a decrease in Cx43 expression is associated with increasing proliferation and a higher tumor grade, but low-grade gliomas (for example Grade II) show increased levels of Cx43 (reviewed in ref. [27]). GJ intercellular communication between glioma cells and endothelial cells is also thought to play a critical role in glioma invasion. Up-regulation of Cx43 in micrometastases of breast cancer appears to facilitate their attachment to the pulmonary endothelium [27]. Thus, it looks like that Cx down-regulation facilitates cancer cell escape from solid tumors, while up-regulation promotes the formation of metastasis.

Valiūnas and colleagues [28] have demonstrated that small RNAs may be delivered through GJs composed of Cx43 but not of Cx26 or Cx32 in HeLa, Mβ16tsA (*wt*), and human mesenchymal stem cells. Also, GJ-dependent transfer of si/miRNAs has been shown to occur between primary cardiac myocytes [29]; human cardiac stem cells and postmitotic myocytes [30]; bone marrow stromal and breast cancer cells [31]; glioma cells [32]; glioma stem cells and MSCs [33]. It is assumed that transfer of small RNAs with high molecular weight via GJs is possible due to rod-shaped morphology of siRNAs, a diameter of which allows their passage through GJs with larger pores [34, 35]. However, the transfer of siRNAs between abutted cells through GJs is under debate so far due to experimental difficulties to reject the pinocytotic pathway of transfer [34]. Our previous study was the first that demonstrated the transfer of double stranded siRNA between remote human laryngeal squamous cell carcinoma (LSCC) cells through open-ended and even through Cx43 GJ-containing TTs [5].

In the present study, we used the HeLa cell model to examine the impact of neural Cxs (Cx36, Cx40, Cx43, Cx45, and Cx47) on the following: 1) cell mobility; 2) formation and properties of TTs; and 3) transfer of siRNA between remote cells through TTs.

## Results

### General properties of TTs between HeLa cells

**Fig. 1:**
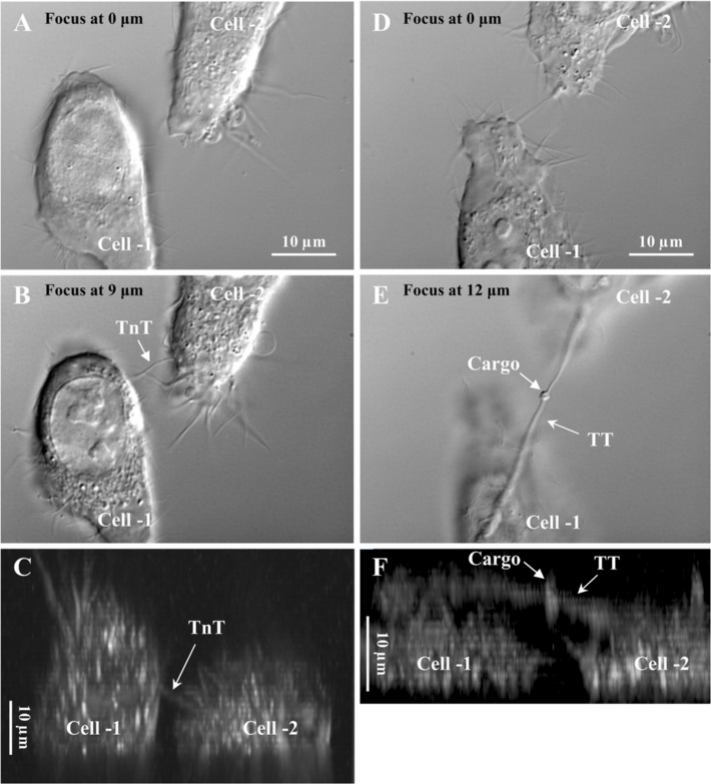
Formation of TTs between HeLa cells. **a-c** TTs formed by the filopodium outgrowth mechanism. **d-f** TTs formed in the process of cell division and successive dislodgment or by the lamellipodium outgrowth mechanism. In both the cases, the pictures represent the top view of cells at a different focus **a** and **b; d** and **e**) and Z-X reconstruction showing TTs raised above the substratum (**c** and **f**)

To examine the impact of different Cx expression on TT morphology, HeLa cells were stably transfected with Cx36-EGFP, Cx40-CFP, Cx43-EGFP, Cx45, or Cx47. Non-transfected HeLa *wt* cells were used as control. We found that HeLa cells, either *wt* or expressing different Cxs, in the culture formed intercellular TTs of various width (ranging from < 200 nm to > 2 μm) and length (up to 70 μm; only TTs longer than 10 μm were taken into account). Time-lapse imaging revealed highly dynamic formation of filopodium-like TTs that were identified as not touching the substratum (Fig. 1a-c). The diameter of the thinnest TTs (<200 nm) could not be measured precisely by conventional optical microscopy as well as their electrical and permeability properties could not be examined due to a short lifetime (tens of seconds).

Much thicker TTs (>300 μm) formed during cell division and subsequent dislodgment or by the lamellipodium outgrowth mechanism. These TTs also were found raised above the substratum (Fig. 1d-f) and were involved in cargo transport either inside the TTs or along their outer surface (indicated by arrows in Fig. 1e and f). However, the leading edges of lamellipodium extensions were usually attached to the substratum and participated in cell motility and TT formation. The lifetime of these TTs lasted tens of minutes and even hours and allowed to use the dual whole-cell patch-clamp technique and fluorescence microscopy for characterization of their formation and properties.

**Fig. 2:**
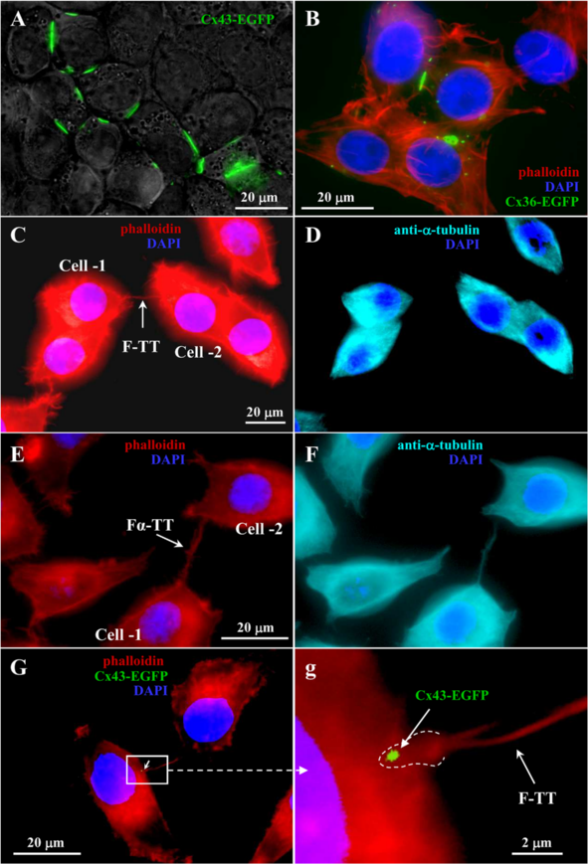
Types of TTs formed between HeLa cells. **a** and **b** A typical view of Cx43-EGFP- and Cx36-EGFP-expressing HeLa cells, respectively, exhibiting multiple fluorescent GJ plaques. **c** and **d** Only F-actin-containing F-TTs. **e** and **f** F-actin- and α-tubulin-containing Fα-TTs. (**G** and **g)** TTs formed GJs at the Cx-expressing cell border (see green dots and a white arrow indicating Cx43-EGFP cluster)

HeLa cells grown to confluence on the glass coverslips formed numerous GJ plaques that can be visible due to chimeric fluorescent proteins (Fig. 2a and b). As it was demonstrated before, abutted HeLa cells expressing Cxs used in the current study formed functional GJs permeable to fluorescent dyes of different molecular weight and net charge [36–38]. In contrast, abutted HeLa *wt* cells did not exhibit any electrical coupling or dye transfer between cells.

However, in this study, the cells were grown at relatively low density and fluorescently tagged proteins helped us confirm the presence and site of GJ plaques in the TT in addition to electrical measurements. We identified two types of TTs between *wt* or different Cx-expressing HeLa cells: TTs containing only F-actin (F-TTs) (Fig. 2c and d) and those containing F-actin and α-tubulin (Fα-TTs) (Fig. 2e and f). The cells were labeled with phalloidin and anti-α-tubulin to visualize the actin network and microtubules, respectively. HeLa cells on average formed 17 and 83 % of F-TTs and Fα-TTs, respectively, and this proportion was not affected by Cxs expression. Cells expressing different Cxs formed TTs with clusters of respective Cxs at the membrane interface with the remote cell (Fig. 2G and g).

### The impact of different Cxs on formation and electrical properties of TTs

**Fig. 3:**
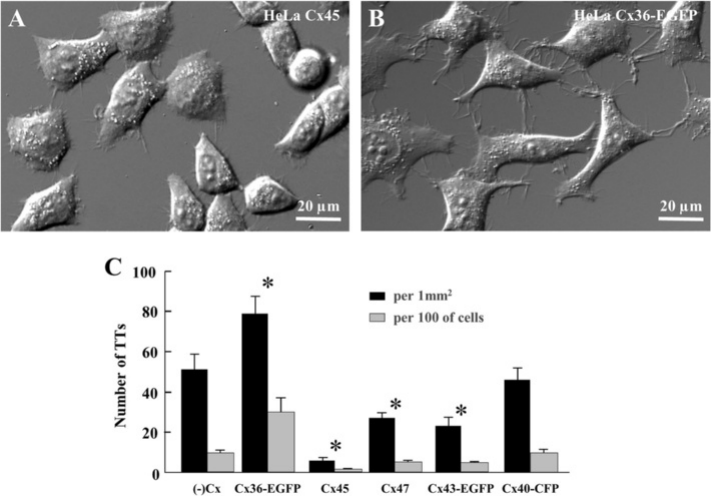
Comparison of TT formation capabilities of HeLa cells expressing different **Cxs.** A typical view of TT formation between HeLa Cx45 (**a**) and Cx36-EGFP cells (**b**). **c** *Wt* and each Cx-expressing HeLa cells were seeded in 24-well plates with glass coverslips on the bottom at equal densities (3 × 10^4^ in each well) and 36 h later were examined using differential interference contrast microscopy with × 20 lens. The experiments were repeated in 2 passages. The number of TTs was counted in 15 randomly selected regions per coverslip and presented as the average values per 1 mm^2^ or per 100 cells (*n* = 2*15 = 30) (see Table 1). *p < 0.05

The properties of TTs between *wt* and Cx-expressing HeLa cells are presented in Table 1. None of these connexins affected the geometry of TTs; however, the number of TTs calculated per 1 mm^2^ or per 100 cells was significantly higher between cells expressing Cx36-EGFP and lower between cells expressing Cx43-EGFP, Cx45, and Cx47 compared with HeLa *wt* cells (Fig. 3).

**Table 1:** Summary of properties of open-ended and GJ-containing TTs

Cx isoform	L (μm)	Number of TTs per mm^2^/ per 100 cells^c^	g_T_ (nS)	siRNA/AF488 permeability (×10^−14^ cm^3^/s)
TT/(−)GJ (HeLa *wt*)	16.3 ± 0.4 (*n* = 214)	51 ± 8/ 9.5 ± 1.3	6.0 ± 1.1^a^ (*n* = 17)	65 ± 38 (*n* = 5)
TT/ Cx36-EGFP	17.0 ± 0.4 (*n* = 330)	79 ± 9^*^/ 30 ± 7.1^*^	0.8 ± 0.2 (*n* = 16)	– ^b^ (*n* = 5)
TT/ Cx40-CFP	15.9 ± 0.5 (*n* = 126)	46 ± 6/ 9.7 ± 1.8	16.5 ± 2.8 (*n* = 20)	4.4 ± 2.0 (*n* = 6)
TT/ Cx43-EGFP	17.0 ± 1.1 (*n* = 96)	23 ± 4^*^/ 4.7 ± 0.7^*^	8.8 ± 3.4 (*n* = 7)	32 ± 17 (*n* = 4)
TT/Cx45	14.3 ± 1.0 (*n* = 68)	5.7 ± 1.6^*^/ 1.4 ± 0.3^*^	4.1 ± 0.5 (*n* = 41)	– ^b^ (*n* = 8)
TT/Cx47	15.1 ± 0.5 (*n* = 112)	27 ± 3^*^/ 5.0 ± 0.9^*^	3.0 ± 0.6 (*n* = 15)	4.3 ± 1.9 (*n* = 6)

“–” nonpermeable; the number of experiments is indicated in parentheses; the diameter of TTs did not depend on the isoform of expressed Cxs and it was 0.9 μm on average (*n* = 210; varied from 0.4 to 2.4 μm)

^*^ *p* < 0.05 compared with HeLa *wt* cells

^a^the majority of junctions were closed-ended (53 out of 70); ^b^ Cx36-EGFP and Cx45 GJs were impermeable to siRNA/AF488; however, they were permeable to AF488 (6.3 ± 1.9 × 10^−14^ cm^3^/s (*n* = 5) and 6.4 ± 4.9 × 10^−14^ cm^3^/s (*n* = 4), respectively); ^c^Cell densities measured 36 h after seeding were 81 ± 5; 75 ± 6; 78 ± 3; 73 ± 7; 70 ± 3; and 79 ± 5 cells per region for *wt*; Cx36-EGFP-; Cx40-CFP-; Cx43-EGFP-; Cx45-; and Cx47-expressing HeLa cells, respectively

**Fig. 4:**
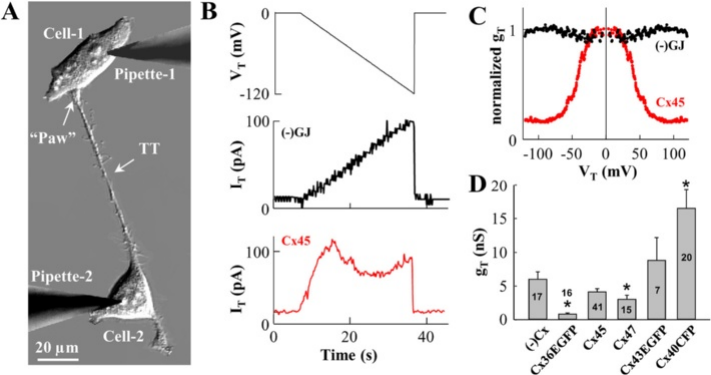
Characterization of electrical properties of TTs formed between HeLa *wt* and Cx36-EGFP-, Cx40-CFP-, Cx43-EGFP-, Cx45-, and Cx47-expressing cells. **a** The TT connecting a pair of HeLa Cx45 cells. **b** Electrical properties of TTs were evaluated by applying a voltage ramp of negative polarity from 0 to −120 mV (**b**, upper panel) to the cell-1 and measuring junctional current in the cell-2 (**b**, middle panel demonstrates a typical I_T_ response of open-ended TTs, and lower panel, of GJ containing TTs); **c** Typical g_T_-V_T_ dependences of open-ended (black) and GJ-containing (red) TTs calculated from I_T_ responses to V_T_ ramps shown in (**b**) with their symmetric counterparts. **d** Summary of conductances of TTs between HeLa *wt* and different Cx-expressing cells (the number of experiments is indicated on the bars; exact numbers are presented in **Table** 1). *p < 0.05

The electrical properties of TTs were examined by the dual whole-cell patch-clamp technique (Fig. 4a). HeLa cells formed functional GJ-containing TTs independent on the isoform of expressed Cx as confirmed by the measurement of voltage gating typical of GJs. Electrical coupling and voltage gating were estimated by applying 30-s voltage ramps from 0 to −120 mV in the cell-1 (Fig. 4b, upper panel) and measuring the current response in the cell-2 (Fig. 4b, middle panel demonstrates the typical I_T_ response of open-ended TTs, and lower panel, of GJ-containing TTs). g_T_-V_T_ dependences (Fig. 4c) were calculated from I_T_ responses to the V_T_ ramps shown in Fig. 4b. Fig. 4d and Table 1 show that g_T_ strongly depended on the single channel conductance of the expressed Cx (presented in the Discussion). Interestingly, g_T_ of open-ended TTs between HeLa *wt* cells was smaller than that between particular Cx-expressing cells. These TTs did not distinguish as having the highest conductance presumably because during cell dislodgment in the process of cytokinesis, open-ended TTs rapidly turn into close-ended ones (~76 % of TTs between HeLa *wt* cells were close-ended) before they rupture. This observation supports the significance of GJs in determining the strength of communication between cells. In Cx-transfected cells, the ratio of close-ended, open-ended, and GJ-containing TTs did not depend on the Cx type and was ~1:2:4 (*n* = 94). In rare cases, TTs between Cx-expressing HeLa cells did not couple the cells electrically for 3 possible reasons: 1) TTs were close-ended; 2) GJ-dependent electrical coupling was not established yet in the process of *de novo* formation of TT; and 3) GJ-dependent electrical coupling was already lost due to cell separation.

In general, g_T_ should at least in part depend directly on TT width (d_T_) and inversely on TT length (L_T_); however, in our experiments g_T_ only moderately correlated with TT geometry suggesting that the total conductance of TTs is more complex. For instance, g_T_ strongly depends on the number of functional GJ channels that are not related to TT geometry and size of GJ plaques [37, 39, 40]. Also, not the external diameter of the TT, but the internal one, is a g_T_-limiting factor, and unfortunately, there are no means for estimation of its dimensions.

### The impact of different connexins on the mobility of HeLa cells

**Fig. 5:**
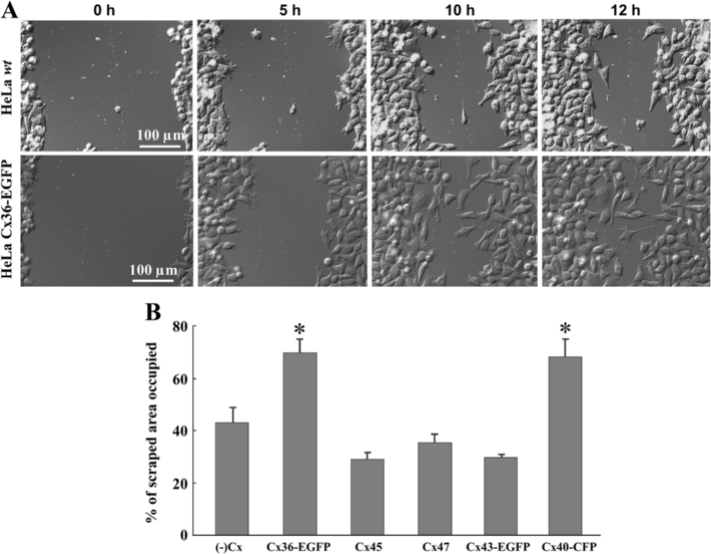
Evaluation of mobility properties of different Cx-expressing HeLa cells by the wound healing assay. **a** The representative pictures of wound healing in HeLa *wt* and HeLa Cx36-EGFP cell monolayers at different time points (5, 10, and 12 h after wound formation). **b** The percentage of the scraped area occupied 12 h after wound formation in different Cx-expressing HeLa cell monolayers (*n* = 3). *p < 0.05 compared with HeLa *wt* cells

The mobility properties of different Cx-expressing HeLa cells were examined by the wound healing assay (Fig. 5). Occupation of the scraped area by Cx36-EGFP- and Cx40-CFP-expressing cells was faster compared with *wt* or Cx43-EGFP-, Cx45-, and Cx47-expressing cells; however, there was no statistically significant difference between *wt* and Cx43-EGFP-, Cx45-, or Cx47-expressing cells. The percentage of the occupied scraped area after 12 h was as follows: in HeLa *wt*, 42.8 ± 6.0 %; HeLa Cx36-EGFP, 69.6 ± 5.4 %; HeLa Cx40-CFP, 68.3 ± 6.7 %; HeLa Cx43-EGFP, 29.7 ± 1.1 %; HeLa Cx45, 29.0 ± 2.4 %; and in HeLa Cx47 cells, 35.3 ± 3.3 %.

### Cx isoform-specific permeability of TTs to siRNA

**Fig. 6:**
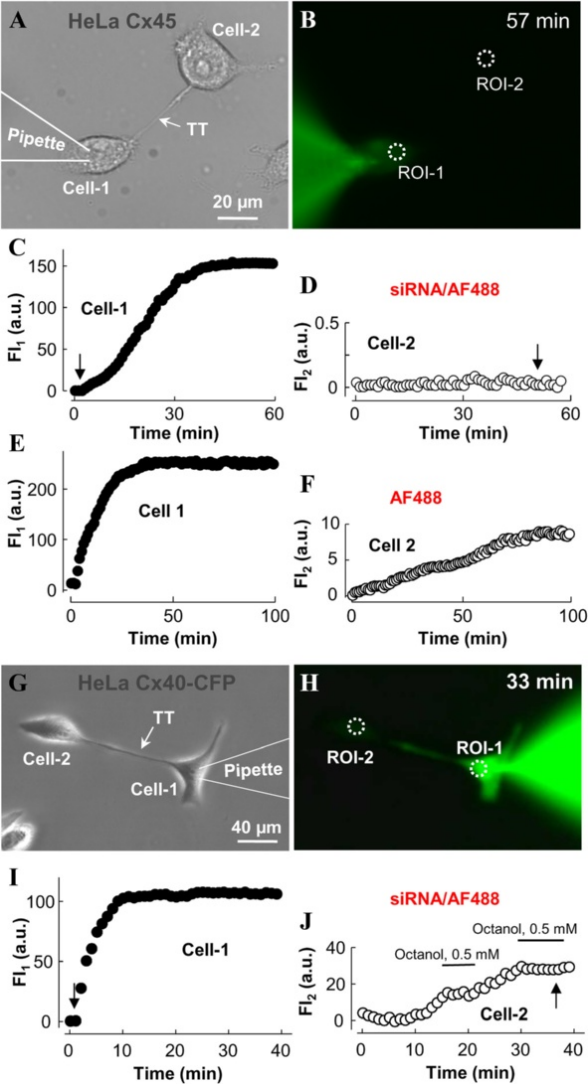
SiRNA transfer through TTs between different Cx-expressing HeLa cells. **a** A pair of Cx45-expressing HeLa cells connected through the TT. **b** SiRNA/AF488 (2 μM) introduced into the patch pipette entered the cell-1 after patch opening, spread along the TT but did not enter the remote cell-2. **c** and **d** Kinetics of siRNA/AF488 accumulation in the cell-1 and the cell-2, respectively. g_T_ measured at the end of the experiment was 2.6 nS. Arrows point to the moments of patch opening in the cell-1 and the cell-2. **e** and **f** Kinetics of AF488 accumulation in the cell-1 and the cell-2, respectively. **g** A pair of Cx40-CFP-expressing HeLa cells connected through the TT. **h** After the patch was opened in the cell-1, siRNA/AF488 entered the cell-1, spread along the TT, and accumulated in the cell-2. **i** and **j** Kinetics of siRNA/AF488 accumulation in the cell-1 and the cell-2, respectively. siRNA/AF488 accumulation in the cell-2 was arrested by octanol (0.5 mM), a GJ blocker. g_T_ measured at the end of the experiment was 10 nS

Previously, we have reported that small RNAs (siRNA/AF488, negative control double stranded siRNA conjugated with AF488) were capable of transiting between LSCC cells through open-ended and Cx43 GJ-containing TTs. In this study, we examined whether TTs containing GJs composed of different neural Cxs were permeable to siRNA using the same approach as described previously [5]. To measure TT permeability, the pipette-1 containing siRNA/AF488 (2 μM) was attached to the cell-1 (Fig. 6a) and after opening the patch, siRNA diffused to the cell-1 followed by its transfer or not through the TT to the cell-2 (Fig. 6b). Typically, accumulation of siRNA/AF488 in the cell-2 started after ~10 min delay compared with cell-1. The total permeability of TT, P_T_, was evaluated using equation 3, which accounted for changes in fluorescence intensity in the cell-1 (FI_1_, Fig. 6c) and the cell-2 (FI_2_, Fig. 6d). At the end of siRNA transfer measurement, the patch in the cell-2 was opened to measure g_T_ and g_T_-V_T_.

As it is demonstrated in Figs. 6a-d, TTs between HeLa cells expressing Cx45 and Cx36-EGFP were impermeable to siRNA/AF488 even though they exhibited substantial electrical coupling and permeability to AF488 (Figs. 6e and f). However, TTs containing Cx40-CFP, Cx43-EGFP, and Cx47 GJs as well as open-ended TTs between non-transfected HeLa *wt* cells were permeable to siRNA/AF488 (Figs. 6g-j, Table 1).

Additional file 1: Figure S1 demonstrates that in the monolayer of HeLa *wt* cells, AF488 injected into the single cell does not spread to the adjacent cells (A-E), and there is no electrical coupling between the abutted HeLa *wt* cells (F and G).

## Discussion

Agnati and his colleagues in their elegant review divided the intercellular communication in the brain into two main modes: wiring transmission (neuronal processes and TTs with their electrical and chemical synapses) and volume transmission (extracellular vesicles) [41]. However, both these modes can be encompassed by TTs that connect cells over long distances by establishing open-ended (direct cell-to-cell channels), close-ended (synaptic transmission and/or active transport), or GJ-based connections. In such a way, TTs are implicated in intercellular electrical and metabolic coupling as well as transfer of vesicles, proteins, organelles, and even genetic material. All these processes are involved not only in the normal functioning of the brain but also in the development and progression of neurodegenerative diseases and brain tumors. Our recent study has demonstrated that TTs containing Cx43 GJs were capable of transferring siRNA/AF488 [5] and raised a question whether TTs containing GJs composed of other neural Cxs are permeable to it. In parallel, we evaluated a GJ channel- and hemichannel-independent impact of the same Cxs on HeLa cell migration and development of TTs.

The novelty and main findings of the current study are the following: 1) Cx36-EGFP promotes while Cx43-EGFP, Cx47, and especially Cx45 inhibit the formation of TTs between HeLa cells; 2) Cx36-EGFP- and Cx40-CFP-expressing HeLa cells demonstrate better mobility properties; 3) TTs containing Cx40-CFP, Cx43-EGFP, and Cx47 are permeable while containing Cx36-EGFP and Cx45 are not permeable to siRNAs.

As it is seen from Table 1, the conductance of GJ-containing TTs depended on the type of the expressed Cx, i.e. Cxs with higher single channel conductance (single channel conductances of Cx36, Cx45, Cx47, Cx43, and Cx40 are ~ 10, 30, 55, 100, and 170 pS, respectively (reviewed in ref. [19])) determined the higher conductance of the TT assuming that the number of channels in the GJ plaque-containing TTs was alike in all cases since TT geometry was not affected by the type of the expressed Cx. Open-ended TTs of HeLa *wt* cells did not distinguish by the highest conductance, presumably because open-ended TTs rapidly turn into close-ended during cell dislodgment in the process of cytokinesis (~76 % of the TTs between HeLa *wt* cells were close-ended). This observation supports the role of GJs in determining the strength of communication between remote cells connected through TTs.

One of the steps in the cell motility cycle is integrin-dependent adhesion to the substrate [42]. Our observation that Cxs localize on the tips of lamellipodium-like protrusions and at their contact with the remote cell suggests that Cxs may interact with cellular adhesion and tight junctional proteins of other cells. For instance, Cx43 has been shown to exert effects on migration by interfering with receptor signaling, cytoskeleton remodeling, and tubulin dynamics [15]; in the developing brain, Cx43 adhesion-promoting properties facilitate the migration of pyramidal cell precursors from the ventricular zone toward the ventricular plate [43, 44]. Also, the expression of Cx43 inversely correlated with the migration rate in the culture of canine brain tumor cells [45]. Similarly, in our study, Cx43-EGFP- as well as Cx45- and Cx47-expressing HeLa cells demonstrated tendency to reduced mobility compared with HeLa *wt* cells (Fig. 5b). Interestingly, cells expressing the same Cxs exhibited reduced formation of TTs while Cx36-EGFP-expressing cells formed the largest number of TTs and demonstrated the highest mobility (Fig. 3c); Cx40-CFP increased cell mobility but TT formation did not differ from that in HeLa *wt* cells. These observations suggest that cell mobility and TT formation may be regulated through different mechanisms that may be controlled by different Cxs in an opposite manner.

The Cx isoform-specific permeability of TTs to siRNA/AF488 suggests that GJs may play an important role in forming the limiting barrier of genetic material transfer through homotypic and heterotypic homocellular and heterocellular connections. TTs containing GJs composed of Cxs with the lowest single channel conductances (Cx36-EGFP and Cx45) were impermeable to siRNA/AF488; however, they both were permeable to AF488 (Fig. 6). The differences in permeability could be determined by different diameters of GJ pores and by distribution of fixed charge sites in the pore. The main factor that can facilitate the effective permeability of genetic material is rod-shaped morphology of a siRNA molecule [34, 35]. Also, it is worth noting that siRNA transfer is not a simple process of diffusion but rather energy-dependent, motor protein-involving transportation. For instance, kinesin and dynein have been shown to be motor proteins associated with microtubule transport [46] that can be blocked by azide, an inhibitor of ATP synthesis [47, 48]. This assumption stays in line with our observation that g_T_ and P_T_ of siRNA did not correlate either among Cxs with different single channel conductances (see Table 1) or in the single Cx series of experiments.

Epithelial-to-mesenchymal and mesenchymal-to-epithelial transitions play crucial roles in cancer metastasis, and these processes can be controlled and reversed by miRNAs [49]. The known pathways of miRNA transfer between cells are the following: extracellular vesicles (exosomes, ectosomes, and apoptotic bodies); circulating RNA in a vesicle-independent form; synapses; GJs and TTs [50]. The last pathway would be the most swift and efficient; however, it still is not definitely proven. A general view that Cxs are down-regulated in cancer cells needs to be revised because many studies including our own [5] have demonstrate the presence of Cxs and GJs in tumor tissues. Even though epithelial-to-mesenchymal transition is associated with Cx down-regulation or loss of communication through GJs between cells that communicate normally (this process is related to the onset of neoplasia and tumorigenesis), mesenchymal-to-epithelial transition is related to the up-regulation of Cx expression and acquisition of novel GJ-based communication between cell types that do not communicate in healthy tissue (these processes are related to angiogenesis, invasion, and metastasis) (reviewed in refs. [12, 51]). For instance, in glioma cell populations, the over-expression of Cxs and GJs between tumor and non-tumor glia cells facilitates the invasion of glioma cells [52]. The expression of Cx43 in the glioma core is very heterogeneous: Cx expression is restricted to minor populations of cells endowed with invasive and cancer stem cell-like properties and able to migrate, but other cells non-expressing Cx43 are able to proliferate [27]. Migrating glioma cells expressing Cx43 may then be able to induce the development of secondary or recurrent gliomas with GJs [13]. Cx43 expression is increased in breast cancer cells predestined to spread to the brain [53]. Disseminating breast cancer or melanoma cells migrate along the luminal surface searching for suitable sites to extravasate and form functional GJs (Cx43, Cx26) with brain endothelial and/or glial cells to initiate brain metastasis, and first lesions develop in Cx-rich vasculature and stroma of the brain [54]. Recent studies have suggested the involvement of miRNA in coordination of the gene expression program determining tumor metastasis [55]. Delivery of miRNA to remote cells can be facilitated by open-ended or GJ-containing TTs. The first evidence of the effectiveness of GJ-dependent pathway was provided by Valiunas and colleagues, who demonstrated that siRNA delivered through GJs down-regulated a reporter gene in the recipient cell [35].

## Conclusions

Our data demonstrate a new modulatory effect of different neural Cxs on cell migration, TT formation, and permeability to siRNA. These results may contribute to the knowledge about mechanisms of cancer invasion and metastasis.

## Methods

### Cell lines and culture conditions

Experiments were performed on HeLa (human cervix carcinoma, ATCC CCL-2, Manassas, VA, USA) cells stably transfected with Cxs tagged with green or cyan fluorescent proteins (Cx36-EGFP, Cx43-EGFP and Cx40-CFP) or untagged Cx45 and Cx47. Stable HeLa cell lines expressing Cxs used in this study were obtained in collaboration with the laboratory of Dr. F. Bukauskas (Albert Einstein College of Medicine, New York, USA). Briefly, vectors were transfected into HeLa cells using Lipofectamine 2000 (Invitrogen, USA) and following the transfection protocol of manufacturer. Cell lines expressing Cx36-EGFP and Cx43-EGFP were selected using 500 μg/ml G418/geneticin (Sigma-Aldrich Co.), whereas 1 μg/ml puromycin (Invitrogen, USA) was used for selection of HeLa Cx40-CFP, Cx45, and Cx47 cell lines. The construction protocols of vectors are described elsewhere [56, 57]. Cells were grown in DMEM medium containing 10 % fetal bovine serum (FBS), penicillin/streptomycin mix (100 U/ml penicillin and 100 μg/ml streptomycin; Gibco Laboratories). Typically, the cells were analyzed on the second day after passage. LSCC cells were prepared as described elsewhere [5].

### Time lapse imaging

Time lapse imaging of HeLa cell mobility and TT formation in the culture medium was performed at 37 °C in the humidified atmosphere of 5 % CO_2_ using an incubation system INUBG2E-ONICS (Tokai Hit, Shizuoka-ken, Japan) with an incubator mounted on the stage of motorized Olympus IX81 microscope (Olympus Europe holding Gmbh, Hamburg, Germany) with Orca-R^2^ cooled digital camera (Hamamatsu Photonics K.K., Japan), fluorescence excitation system MT10 (Olympus Life Science Europa Gmbh, Hamburg, Germany), and XCELLENCE software (Olympus Soft Imaging Solutions Gmbh, München, Germany).

### Wound healing assay

Cell migration analysis was performed by the wound healing assay. Cells were grown to confluence on the glass coverslips. Then the monolayer was scraped with a sterile surgical blade. The cells were washed with fresh growth medium to remove cell debris. Wound healing was evaluated by measuring the cell-free area remaining in the wound [58] after 5, 10, or 12 h.

### Electrophysiological measurements

For simultaneous electrophysiological and fluorescence recording, cells grown onto glass coverslips were transferred to an experimental chamber with constant flow-through perfusion mounted on the stage of the inverted microscope Olympus IX8. Junctional conductance g_T_ between the cells connected by the TT was measured using the dual whole-cell patch-clamp technique. Cell-1 and cell-2 of a cell pair were voltage clamped independently with a patch-clamp amplifier MultiClamp 700B (Molecular Devices, Inc., USA) at the same holding potential, V_1_ = V_2_. Voltages and currents were digitized using a Digidata 1440A data acquisition system (Molecular Devices, Inc., USA) and acquired and analyzed using pClamp 10 software (Molecular Devices, Inc., USA). By stepping the voltage in the cell-1 (ΔV_1_) and keeping the other constant, junctional current was measured as the change in current in the unstepped cell-2, I_T_ = ΔI_2_. Thus, g_T_ was obtained from the ratio -I_T_/ΔV_1_, where ΔV_1_ is equal to transjunctional voltage (V_T_) and negative sign indicates that the junctional current measured in cell-2 is oppositely oriented to the one measured in cell-1. To minimize the effect of series resistance on the measurements of g_T_ [59], we maintained pipette resistances below 3 MOhms. Patch pipettes were pulled from borosilicate glass capillary tubes with filaments. Experiments were performed at room temperature in modified Krebs-Ringer solution (in mM): NaCl, 140; KCl, 4; CaCl_2_, 2; MgCl_2_, 1; glucose, 5; pyruvate, 2; HEPES, 5 (pH 7.4). Patch pipettes were filled with saline containing (in mM): KCl, 130; Na aspartate, 10; MgATP, 3; MgCl_2_, 1; CaCl_2_, 0.2; EGTA, 2; HEPES, 5 (pH = 7.3).

### Fluorescence Imaging and siRNA Transfer Studies

Fluorescence signals were acquired using the Olympus IX81 microscope with Orca-R^2^ digital camera, fluorescence excitation system MT10, and XCELLENCE software. For siRNA transfer studies, siRNA conjugated with Alexa Fluor-488 fluorescent dye (siRNA/AF488, QIAGEN, Venlo, Netherlands) or Alexa Fluor-488 hydrazide (AF488, Life Technologies) was introduced into cell-1 of a pair through a patch pipette in whole-cell voltage-clamp mode. Typically, this resulted in loading of the cell-1, followed by siRNA/AF488 or AF488 transfer via the TT to the neighboring cell-2. At the end of siRNA/AF488 or AF488 transfer measurement, the patch in the cell-2 was opened to measure g_T_ in dual whole-cell patch-clamp mode. The presence or absence of GJ in the TT was checked by measuring g_T_-V_T_. Evaluation of GJ permeability to fluorescent dyes from changes in fluorescence intensity in both cells was previously described elsewhere [36, 39, 40]. In brief, the cell-to-cell flux (J_T_) of the dye in the absence of transjunctional voltage (V_T_ = 0 mV) can be determined from changes of dye concentration in the cell-2 (ΔC_2_) over the time interval (Δt) as follows:

1 $$ {J}_T=\frac{vo{l}_2\cdot \varDelta {C}_2}{\varDelta t} $$

where vol_2_ is the volume of cell-2. Then, according to the modified [60] Goldman-Hodgkin-Katz (GHK) equation [61], the total junctional permeability (P_T_) can be described in consequence:

2 $$ {P}_T=\frac{J_T}{C_1-{C}_2}=\frac{vo{l}_2\cdot \varDelta {C}_2}{\varDelta t\cdot \left({C}_1-{C}_2\right)} $$

where C_1_ and C_2_ are dye concentrations in the cell-1 (dye donor) and the cell-2 (dye recipient), respectively. Cell volume was approximated as a hemisphere. The diameter of a hemisphere was determined by averaging the longest and the shortest diameters of the cell; the volume of examined HeLa cells was ~1800 μm^3^ on average. Assuming that the dye concentration is directly proportional to fluorescence intensity (C = k ▪ FI), equation 2 can be modified as follows:

3 $$ {P}_T=\frac{vo{l}_2\cdot \varDelta F{I}_2}{\varDelta t\cdot \left(F{I}_1-F{I}_2\right)} $$

where ΔFI_2_ = FI_2,n+1_ - FI_2,n_ is the change in FI in cell-2 over time, Δt = (t_n+1_ - t_n_); n is the nth time point in the recording. To minimize siRNA/AF488 bleaching, studies were performed using time-lapse imaging, which exposed cells to low-intensity light for ~0.5 s every 1 min.

### Immunocytochemistry of cells

Cells were grown in 24-well plates with glass coverslips on the bottom, fixed with 4 % paraformaldehyde for 15 min, and permeabilized with 0.2 % Triton X-100/PBS for 3 min. Coverslips were incubated for 1 h with mouse anti-α-tubulin (Sigma-Aldrich, Steinheim, Germany) primary antibody, then rinsed with 1 % BSA/PBS and incubated for 30 min with goat anti-mouse IgG H&L (Cy5) (Abcam Cambridge*,* UK) secondary antibody. The F-actin network was visualized using Alexa Fluor 594 phalloidin (Invitrogen, USA), coverslips were incubated with the dye for 30 min at 37 °C. Analysis was performed with the Olympus IX81 microscope equipped with Orca-R^2^ digital camera, fluorescence excitation system MT10, and XCELLENCE software.

### Data analysis and statistics

The analysis was performed using SigmaPlot software (Systat, Richmond, CA, USA), and averaged data are reported as means ± SEM. For statistical evaluation, the Student’s *t* test was used, and a difference was considered statistically significant when p was < 0.05.

## Additional file

Additional file 1: Figure S1. Dye permeability and electrical coupling between HeLa *wt* cells. (A-C) AF488 was introduced into a single cell (ROI-1) of the HeLa *wt* cell monolayer, and the time-lapse imaging of fluorescence intensity was monitored in the surrounding cells (ROI-2 – ROI-6). (D and E) Kinetics of AF488 accumulation in the injected cell and neighboring cells, respectively (background fluorescence subtracted) (*n* = 6). The arrow indicates the moment of patch opening. Circles filled with different colors in E represent FI from ROI-2 – ROI-6. (F-G) Electrical coupling of abutted HeLa *wt* cells was evaluated by applying voltage ramp (V_j_) of negative polarity from 0 to −120 mV (G, upper panel) to the cell-1 and measuring junctional current (I_j_) (G, lower panel) in the cell-2 (*n* = 17). (TIF 1708 kb)

## Abbreviations

TT: tunneling tube
F-TT: tunneling tube containing only F-actin
Fα-TT: tunneling tube containing F-actin and α-tubulin
C: concentration of the dye
Cx: connexin
FI: fluorescence intensity of the dye
GJ: gap junction
g_j_: junctional conductance of abutted cells
g_T_: conductance of the TT
I_T_: current through the TT
J_T_: cell-to-cell flux of the dye
P_T_: permeability of the TT
siRNA: small interfering RNA
V_j_: transjunctional voltage of abutted cells
V_T_: voltage across the TT
LSCC: human laryngeal squamous cell carcinoma
