# Supplementary figures and images for: The role of neural connexins in HeLa cell mobility and intercellular communication through tunneling tubes
Source: BMC Cell Biol. 2016 Jan 13;17:3. doi: 10.1186/s12860-016-0080-1 (PMC4710989; doi:10.1186/s12860-016-0080-1)

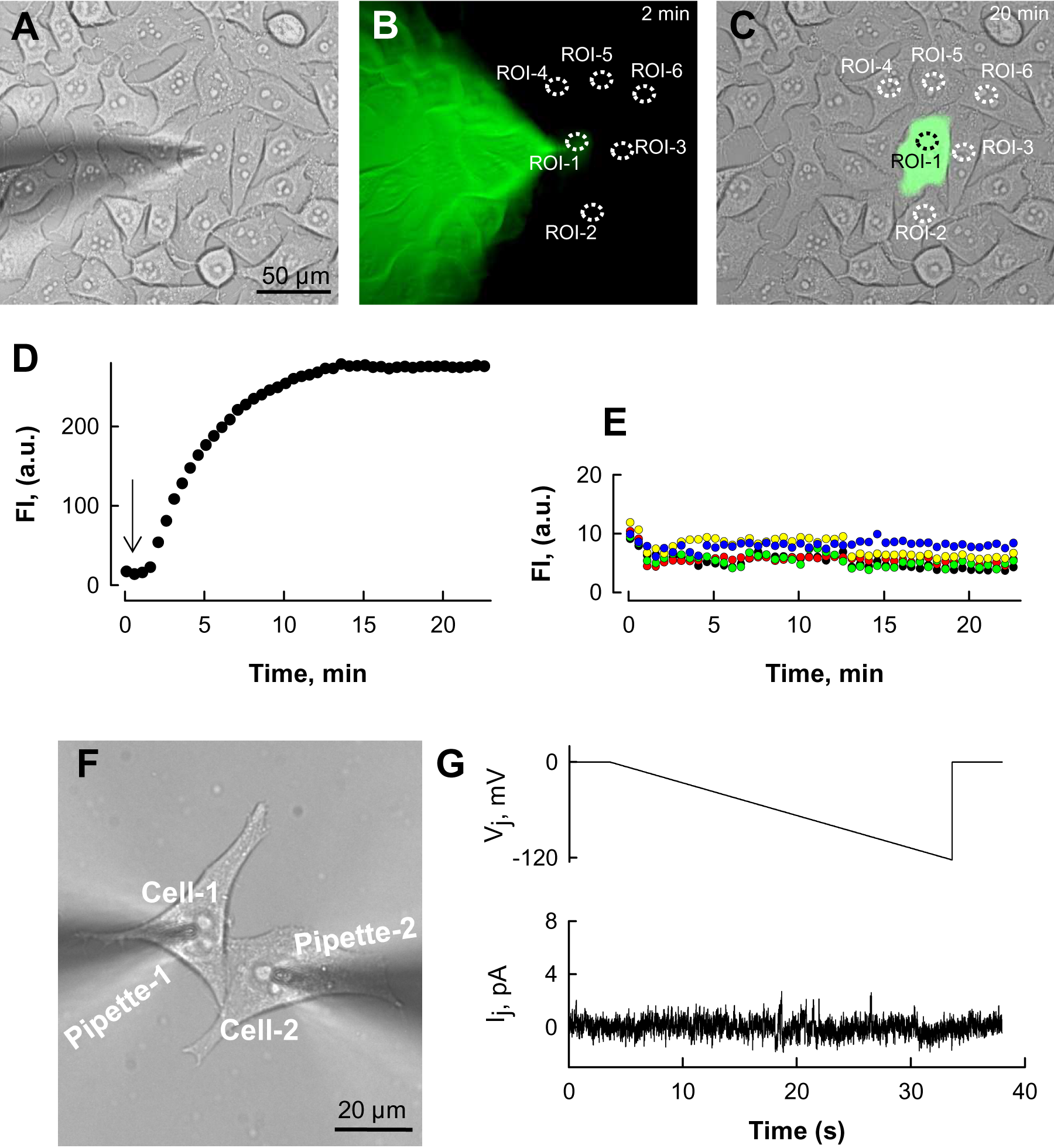

Supplement: Additional file 1: Figure S1. — Dye permeability and electrical coupling between HeLa wt cells. (A-C) AF488 was introduced into a single cell (ROI-1) of the HeLa wt cell monolayer, and the time-lapse imaging of fluorescence intensity was monitored in the surrounding cells (ROI-2 – ROI-6). (D and E) Kinetics of AF488 accumulation in the injected cell and neighboring cells, respectively (background fluorescence subtracted) (n = 6). The arrow indicates the moment of patch opening. Circles filled with different colors in E represent FI from ROI-2 – ROI-6. (F-G) Electrical coupling of abutted HeLa wt cells was evaluated by applying voltage ramp (Vj) of negative polarity from 0 to −120 mV (G, upper panel) to the cell-1 and measuring junctional current (Ij) (G, lower panel) in the cell-2 (n = 17). (TIF 1708 kb) [file 12860_2016_80_MOESM1_ESM.tif]
